# Polygenic risk scores in human genetics for study design discovery and translation

**DOI:** 10.1007/s00439-026-02873-y

**Published:** 2026-09-19

**Authors:** Hui-Qi Qu, Hakon Hakonarson

**Affiliations:** 1 The Center for Applied Genomics, Children’s Hospital of Philadelphia, 3615 Civic Center Blvd, Abramson Building, Philadelphia, PA 19104 USA; 2 Department of Pediatrics, The Perelman School of Medicine, University of Pennsylvania, Philadelphia, PA 19104 USA; 3 Division of Human Genetics, Children’s Hospital of Philadelphia, Philadelphia, PA 19104 USA; 4 Division of Pulmonary and Sleep Medicine, Children’s Hospital of Philadelphia, Philadelphia, PA 19104 USA; 5 Faculty of Medicine, University of Iceland, 101 Reykjavik, Iceland

## Abstract

Polygenic risk scores (PRS) quantify the component of disease risk captured by measured additive common variants. Their use as a study design variable, rather than only as a predictive endpoint, broadens their scientific value in human genetics. PRS can define informative extremes of common-variant burden, identify discordance between phenotype and PRS-estimated risk, and facilitate the detection of subgroup-specific or residual mechanisms that may be obscured in conventional case-control analyses. This review outlines four major PRS-informed design strategies: tail sampling based on PRS extremes; discordance sampling based on mismatch between phenotype and PRS-implied risk; conditional and stratified genome-wide association analyses using PRS to adjust for / partition background risk; and residual phenotype analysis of the component of phenotype remaining after the PRS-associated component has been removed. It thus provides a practical framework for sample enrichment, subgroup definition, and calibrated epidemiologic comparison. These designs may be especially informative when integrated with sequencing, multi-omics, longitudinal cohorts, and translationally oriented intervention studies. Their application requires careful attention to data leakage, ancestry-related bias, collider structures, and the limits of interpretation.

## Introduction

Polygenic risk scores (PRS) entered human genetics as prediction tools after aggregation of common-variant effects was shown to capture susceptibility distributed across many loci (Purcell et al. 2009; Wray et al. 2007). PRS then became concise summaries of inherited susceptibility and were judged mainly by discrimination, variance explained, and clinical utility (Chatterjee et al. 2016; Lewis and Vassos 2020; Torkamani et al. 2018). That predictive framing was productive, but it left another use of PRS less fully developed. PRS is a measurable summary of common-variant risk captured by existing genome-wide association studies (GWAS), which makes it useful for study design and influences downstream processes.

The design question differs from the prediction question. Prediction asks how well a score ranks or calibrates risk in individuals or populations. Design asks whether a score captures enough information, here known as common-variant risk, to help select samples and structure comparisons that influence downstream analytical approaches. A PRS need not be clinically predictive to be useful for discovery-oriented design, because it can still measure part of the background risk relevant to sampling and comparison. Its limits may also help define what remains to be found.

This perspective is especially relevant because human complex disease is biologically heterogeneous. Standard pooled case-control analysis is often efficient for detecting shared effects, but it can blur or dilute mechanisms present only in subsets of patients, or mechanisms that become visible only after known polygenic background has been accounted for. PRS offers a practical way to measure that background and its use in study design with translational implications.

### The predictive framing of PRS

PRS work has centered on risk stratification, including screening, prognosis, prevention, and clinical translation (Lewis and Vassos 2020; Torkamani et al. 2018; Wand et al. 2021). PRS have also been used to model treatment response (Zhai et al. 2022) and quantify shared genetic risk across related phenotypes, such as psychiatric disorders like schizophrenia and bipolar disorder (Purcell et al. 2009). For some common diseases, individuals in the extreme upper tail of a genome-wide PRS distribution can have risk comparable to that associated with certain monogenic variants (Khera et al. 2018). In the literature, PRS is usually treated as the final product: a predictor to validate, calibrate, and potentially deploy.

That emphasis shaped how success was measured. PRS studies are commonly evaluated by area under the receiver operating characteristic curve (AUC), R^2^, calibration, or odds ratios with confidence intervals. These metrics are appropriate when the goal is risk prediction. They are less informative when the goal is discovery-oriented study design. A score can still be useful in discovery settings because design utility depends on whether it captures enough common-variant risk to support informative sampling and comparison.

### PRS as a summary of common-variant risk

For design purposes, PRS is best understood as a model-dependent, population-specific estimate of part of additive common-variant risk. Under the liability-threshold model, disease develops when an unobserved liability exceeds a threshold (Falconer 1967). PRS does not recover total risk. It captures the component represented by the training GWAS, variant set, scoring method, and target-population calibration (Choi et al. 2020; Dudbridge 2013). Its performance and interpretation are also affected by ancestry, ascertainment, imputation quality, and reporting transparency (Duncan et al. 2019; Wand et al. 2021). Among these factors, genotyping technology is particularly important because it influences which variants enter GWAS discovery and subsequent PRS construction.

PRS should be interpreted as a model-specific estimate of inherited susceptibility rather than as a broad measure of genetic risk. It measures one defined part of inherited susceptibility represented by the additive common-variant model used to build the score. Everything outside that fitted model remains outside the score. The gap between observed phenotype and PRS-estimated risk may reflect rare variants, structural variation, non-additive effects, environmental exposures, developmental processes, or phenotype misclassification. Examples include interaction between *HLA-DR/DQ* haplotypes in type 1 diabetes (Sharp et al. 2019) and monogenic diabetes misdiagnosed as type 1 diabetes. That gap is not just residual noise. It can help define sources of risk not captured by the fitted PRS model.

### From prediction to design

Depending on the scientific question, PRS can be used to select informative individuals, identify phenotype-risk mismatch, structure association analysis, or define a residual outcome for downstream study (Chatterjee et al. 2016; Choi et al. 2020; Zhou et al. 2021). In these settings, the score structures the comparison rather than simply ranking risk. We refer to this broader use as PRS-informed design, in which PRS guides sampling, subgroup definition, and analytic comparison for discovery-oriented and translational studies.

The relevant polygenic background is not always best represented by one global score. Context-specific PRS defined by sex, age, ancestry, pathway, exposure, cell type, or another biologically meaningful axis may better align the score with the mechanism under study (Jamialahmadi et al. 2024; Qu et al. 2026; Schork and Elman 2023). However, this increased specificity can reduce effective sample size, increase multiple-testing burden, and limit feasibility in smaller cohorts or clinical trials.

**Fig. 1:**
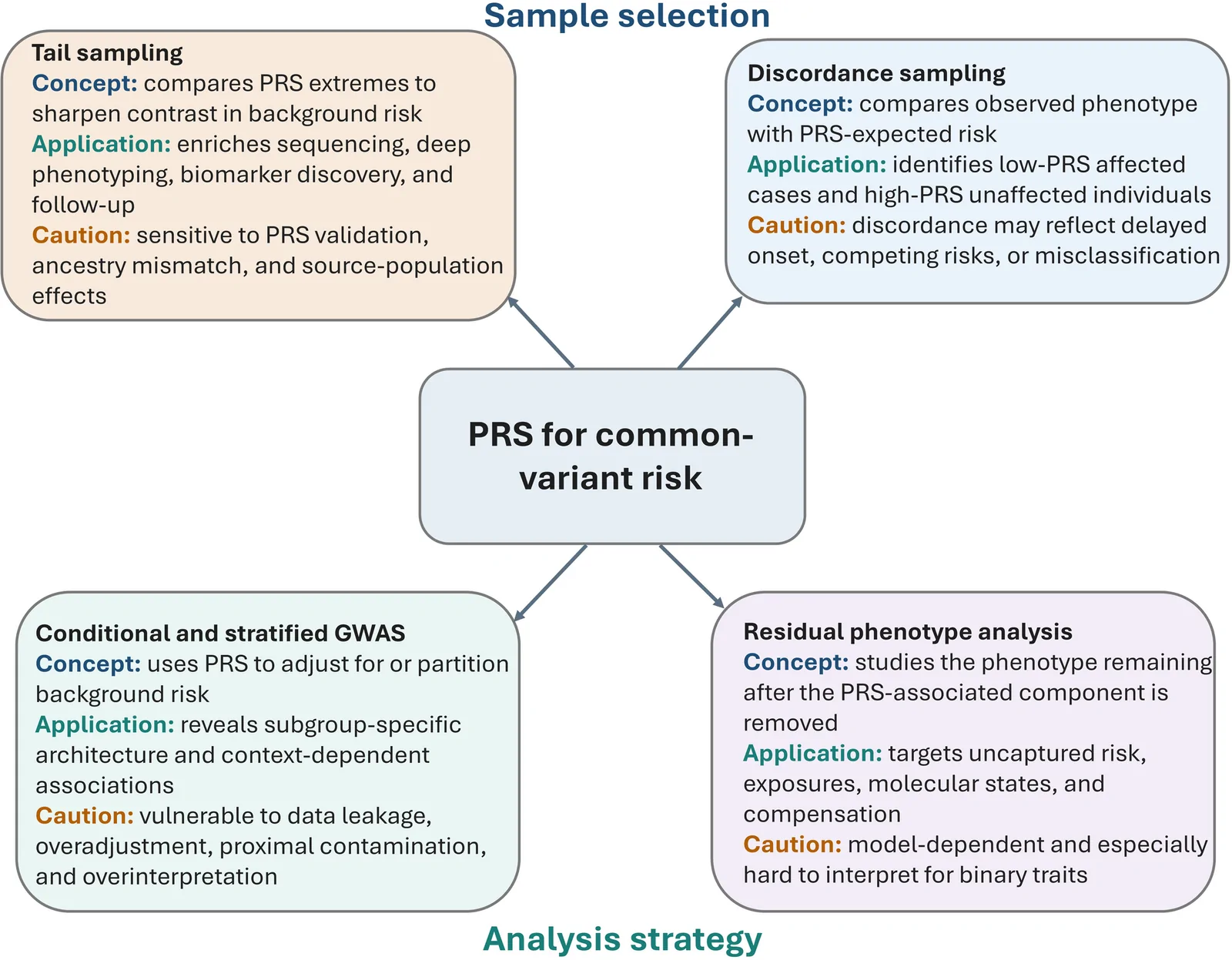
Four major PRS-informed design strategies. These strategies can be applied separately or in combination, and their boundaries may overlap within a single empirical study. Rather than functioning as mutually exclusive categories, they represent complementary ways of using PRS. For example, a study may use PRS to define an informative subgroup, structure association testing, and interpret the phenotypic variation that remains after accounting for PRS-associated risk. Interpretation depends on score validity, target-population validation, avoidance of data leakage, and separation of enrichment from causal inference

Four major strategies (Fig. 1) follow from this perspective, including tail sampling, PRS discordance sampling, conditional and stratified GWAS, and residual phenotype analysis. They differ mainly in what PRS is accomplishing: selecting individuals, identifying mismatch between phenotype and modeled risk, structuring association analysis, or redefining the outcome after accounting for PRS. The same empirical study may use more than one of these approaches.

## A general framework for expected risk and informative deviation

The design value of PRS becomes clearer when liability is written schematically as a PRS-captured component plus a remainder. A useful representation is:

$$ L{\text{ }} = {\text{ }}L_{{PRS}} + {\text{ }}L_{{otherG}} + {\text{ }}E{\text{ }} + {\text{ }}\varepsilon $$

where ***L*** denotes total latent liability; ***L***_***PRS***_, liability captured by the PRS; ***L***_***otherG***_, inherited susceptibility not captured by the PRS; ***E***, environmental and other non-genetic influences; and ***ε***, stochastic and measurement-related variation.

This model is schematic rather than identifiable from observed data alone. Its value is conceptual and design-oriented rather than strictly parametric. It clarifies what PRS does and does not measure. Once the PRS-captured component is made explicit, the remainder becomes a direct object of study. A central limitation of PRS-informed design is that selecting an informative subgroup can change the associations observed within that subgroup. A low-PRS case series, a high-PRS unaffected group, or a cohort restricted by disease progression is not a random subset of the source population. These designs can sharpen contrasts for discovery, but they may also induce collider or index-event bias and distort observed associations or effect sizes (Mitchell et al. 2023; Munafò et al. 2018).

### Liability decomposition

Under the liability-threshold model, disease reflects a continuous latent liability on which genetic and non-genetic influences accumulate until a threshold is crossed (Falconer 1967). Observed disease status is therefore shaped by multiple contributors: PRS-captured risk, inherited susceptibility not captured by PRS, environment, developmental or stochastic variation, and measurement noise. PRS captures only part of inherited risk, because its performance depends on the training GWAS, scoring model, marker selection, ancestry, and target population (Dudbridge 2013; Duncan et al. 2019). The remaining inherited component may include rare coding variants, structural variants, poorly tagged common variants, ancestry-specific effects, and non-additive architecture not represented by standard PRS models. In particular, application of PRS across ancestry groups requires careful allele harmonization and population-specific validation, because allele frequencies, linkage disequilibrium patterns, imputation quality, and major/minor allele status may differ across populations (Duncan et al. 2019; Martin et al. 2019). Environmental contributors include exposures, infection, behavior, treatment, and social context. Measurement-related variation includes diagnostic misclassification, temporal instability of phenotype, and technical error.

PRS-adjusted or PRS-stratified analysis distinguishes the part already captured by the fitted additive common-variant model from everything outside that model. The remainder may still be genetic, but of a different kind. PRS-captured risk is scientifically important because it provides a measurable and reproducible baseline of additive common-variant susceptibility at population scale. The architecture summarized by PRS reflects signals repeatedly detected across large GWAS and therefore supplies a practical reference. Once that component is measured, investigators can ask whether disease patterns, subgroup differences, or downstream associations are consistent with known common-variant burden or instead point to sources of risk not captured by the fitted PRS model. This distinction is the conceptual basis for treating phenotype-PRS deviation as an object of study.

### What makes a sample informative

A sample is informative when it sharpens contrast for the mechanism under study. Individuals sampled from opposite PRS tails differ more in modeled common-variant burden than randomly sampled individuals. If the score is appropriate for the target population, that stronger contrast can improve the efficiency of discovery.

Affected individuals with low PRS and unaffected individuals with high PRS are informative for another reason, as the observed phenotype and modeled risk are misaligned. Such mismatch may reflect mechanisms not captured by the fitted common-variant model, exposure differences, delayed onset, competing risks, buffering processes, or measurement error.

PRS can also reduce background heterogeneity. Restricting analysis to a narrower PRS range can make individuals more comparable with respect to measured polygenic burden. That does not remove all confounding, but it can reduce dilution from broad susceptibility differences.

### Scientific functions of PRS-informed design

The four main functions of PRS-informed design are distinct. Enrichment uses PRS to recruit individuals from informative parts of the risk distribution before sequencing, deep phenotyping, or follow-up studies are performed. The goal is efficient discovery rather than prediction. Model-referenced comparison uses PRS as a reference for expected common-variant risk against which observed phenotype is compared. PRS provides a model-based estimate of expected common-variant risk (Chatterjee et al. 2016; Dudbridge 2013). Discordance designs apply this framework by defining high-PRS unaffected individuals and low-PRS affected individuals relative to model-based expectation rather than by phenotype alone. Partitioning uses PRS to divide a cohort into risk strata so that association analysis can be performed within or across strata. Residual targeting models phenotype on PRS and then treats the remainder as the analytic outcome. That residual is the component of the analyzed phenotype that remains after the fitted PRS-associated component has been accounted for.

PRS-informed design can sharpen contrast, improve the efficiency of sequencing or deep phenotyping, and help reveal subgroup-specific or residual mechanisms that pooled analysis may obscure. At the same time, comparisons created by PRS-based sampling, stratification, or adjustment should not be interpreted too quickly as unbiased causal estimates. The same study may therefore gain substantially in enrichment and characterization of etiologic heterogeneity while becoming less straightforward for causal interpretation and downstream translational efforts.

## Major PRS-informed design strategies

### Tail sampling for informative extremes

Tail sampling recruits individuals from the extremes of PRS-defined common-variant risk. This design is related to recall-by-genotype sampling, in which genetic information is used to select individuals for detailed phenotyping or follow-up (Corbin et al. 2018). When the score captures a meaningful fraction of inherited susceptibility, PRS tails identify individuals with substantially different modeled polygenic backgrounds, which can support risk enrichment and prevention-oriented follow-up (Khera et al. 2018; Torkamani et al. 2018).

#### PRS extremes are not the same as phenotype extremes

Phenotype extremes and PRS extremes are not interchangeable. Extreme phenotypes often reflect a mixture of inherited burden, environment, treatment, timing, and measurement error. By contrast, PRS tails isolate the component captured by additive common-variant signal (Chatterjee et al. 2016). This is why tail sampling does not compete with extreme-phenotype designs. It answers a different question by selecting on modeled inherited background rather than on observed clinical severity.

#### Design variants

The simplest tail design compares the top and bottom PRS tails. A top-tail-only design may be preferable when the target is high modeled inherited burden, increased event rate, or prevention-oriented follow-up (Khera et al. 2018). Tail definitions can also be implemented by quantile bins rather than by a single cutoff. For example, PRS may be divided into deciles, with analyses comparing the lowest and highest deciles or using the full decile structure to assess gradient effects across the risk distribution (Qu et al. 2021c). This approach makes tail selection more explicit and can clarify whether observed signals are concentrated in the extremes or change more continuously across PRS strata. Internally defined deciles or high/low groups describe relative position within the study sample and should not be treated as absolute low- or high-risk categories. When a PRS from a large GWAS is applied to a smaller cohort, cutoffs should be pre-specified from an external or target-population reference distribution and then applied unchanged (Lennon et al. 2024; Wand et al. 2021). In clinically enriched cohorts, internal cutoffs may improve balance for within-sample comparisons but may not correspond to the same risk percentiles in the source population. Broad pre-specified strata may be preferable to narrow tail contrasts; alternatively, PRS can be modeled continuously, with quantile groups used for sensitivity analysis or visualization. Regression discontinuity designs may be informative when a fixed PRS threshold determines study assignment, to evaluate whether threshold-based assignment changes the downstream outcome or study yield (Cattaneo and Titiunik 2022).

A more controlled design uses phenotype-matched PRS tails, in which individuals with similar phenotype values are sampled from opposite PRS strata. This can separate observed trait level from modeled common-variant risk. Family history can refine these designs further. PRS and family history are related but not interchangeable; across many diseases they provide partly independent information (Mars et al. 2022). Combined use can enrich inherited susceptibility more effectively than either measure alone, but it can also complicate interpretation when the two measures are correlated or capture overlapping sources of risk.

#### Practical constraints

The validity of tail sampling depends on the validity of the PRS used to assign individuals to tails. This requires external derivation or cross-fitting when possible, validation in the target population, and attention to technical platform differences that may shift individuals across relevant thresholds (Ho et al. 2025; Lennon et al. 2024). In-sample optimization can distort tail assignment through overfitting and winner’s curse (Shi et al. 2016). Portability is also critical, because ancestry mismatch can misclassify tails and amplify bias (Martin et al. 2019).

Ascertainment matters as well. A PRS tail defined in a hospital-enriched or case-enriched cohort is not equivalent to the same percentile in a population-based sample. Score distribution, disease prevalence, and the meaning of “extreme” depend on the source population. When the target is population-based inference, thresholds should ideally be calibrated in a population-based reference sample rather than imported directly from an ascertained case-control cohort.

#### Two-dimensional sampling

A useful extension samples jointly on PRS and a second dimension relevant to inherited risk or disease context (Table 1). One version cross-classifies individuals by PRS level and disease status, generating groups such as high-PRS cases, high-PRS controls, low-PRS cases, and low-PRS controls. Another version combines PRS with family history, age at onset, exposure status, or biomarker profile. The value of this design is that it enriches samples along two partially distinct axes rather than one, which can sharpen contrasts for downstream sequencing, phenotyping, or subgroup analysis. Compared with sampling on PRS alone, two-dimensional designs can better distinguish individuals with similar polygenic burden but different familial, environmental, or clinical backgrounds. A limitation is that adding a second sampling dimension can reduce subgroup size and complicate interpretation, especially when the two variables are correlated or capture overlapping sources of risk. In smaller clinical cohorts or trials it may produce sparse cells and unstable subgroup estimates. The design should therefore specify the primary contrast in advance, collapse the second dimension into broad categories when needed, and avoid interpreting underpowered cells as evidence of absence. When sample size is limited, PRS can be modeled continuously while the second dimension is used for adjustment or sensitivity analysis rather than for full stratification.

**Table 1:** Pairwise PRS-tail contrasts and their main uses

Contrast/design	What is good for	Main caution
High-PRS cases vs. high-PRS controls	Penetrance modifiers; delayed onset; candidate protective or compensatory factors	Controls may be preclinical or later-onset cases
Low-PRS cases vs. low-PRS controls	Rare variants; mechanisms not captured by the dominant common-variant model; atypical disease processes; exposure-related risk	Misclassification or poor PRS portability may mimic signal
High-PRS cases vs. low-PRS cases	Risk-dependent biology; case heterogeneity; subtype architecture; contrast between canonical and low-PRS disease mechanisms	Case groups may differ in severity, age at onset, or ascertainment
High-PRS controls vs. low-PRS controls	Risk-state profiling; preclinical biomarkers; prevention enrichment; delayed onset; candidate compensatory biology	Differences may reflect future disease; follow-up is essential
Low-PRS cases vs. high-PRS controls	Extreme discordance; disease mechanisms outside the dominant PRS model versus absence of disease despite high modeled risk	Mechanistic interpretation is less clean
High-PRS cases vs. low-PRS controls	Maximal contrast; biomarker discovery; proof-of-principle	Strong contrast, limited specificity

### PRS discordance sampling

PRS discordance sampling focuses on individuals whose phenotype is not well aligned with PRS-estimated common-variant risk, especially low-PRS affected individuals and high-PRS unaffected individuals.

#### Defining discordance in model-based terms

Discordance is a mismatch between observed phenotype and the common-variant risk estimated by PRS, while PRS only estimates part of common-variant risk rather than total disease risk (Dudbridge 2013). It is informative because it identifies individuals who differ from what the fitted common-variant model would lead us to expect. Discordance depends on the PRS model, the population in which the score is applied, and the quality of phenotype definition.

#### Sources of phenotype-PRS discordance

Discordance can arise from several sources. In low-PRS affected individuals, it may indicate risk that is not well captured by the fitted common-variant model, whether because of rare genetic effects, nonstandard disease mechanisms, phenotype heterogeneity, environmental exposure, or measurement error. In high-PRS unaffected individuals, it may reflect delayed onset, absence of relevant exposure, buffering mechanisms, competing risks, survival effects, or phenotype misclassification. Discordance therefore supports enrichment and heterogeneity discovery, but it does not by itself establish mechanism.

#### Protection, resistance, and compensation

Favorable discordance should not be treated as one biological state. Protection is the broadest term and refers to reduced or absent disease despite expected risk. Resistance is stricter and implies that the upstream pathogenic process is present but disease does not emerge. Compensation implies that pathogenic biology is active but downstream consequences are buffered. These distinctions correspond to different biological hypotheses and different study designs.

Cross-sectional mismatch alone cannot distinguish them. Evidence for resistance requires showing that the upstream pathogenic process is present but does not progress. Evidence for compensation requires showing that pathogenic biology is active but its consequences are attenuated. Longitudinal follow-up, exclusion of preclinical disease, and careful phenotype validation are therefore necessary before applying these labels (Hess et al. 2021; Hou et al. 2022).

#### Low-PRS cases and high-PRS controls are not symmetric

Low-PRS cases and high-PRS controls should not be treated as opposite versions of the same discordance pattern. Low-PRS cases are primarily informative about disease that is not well captured by the dominant common-variant model, whereas high-PRS controls are primarily informative about non-expression, delayed onset, or modification of disease despite high modeled risk (Hess et al. 2021; Lu et al. 2021). The relevant biological questions therefore differ across the two groups, and study design, phenotype validation, and downstream analysis should be tailored accordingly.

#### Longitudinal necessity

High-PRS unaffected status should be interpreted cautiously, especially in age-dependent or slowly progressive disorders, because some individuals classified as unaffected will later become cases (Eissman et al. 2023; Hou et al. 2022). With Type 2 diabetes as an example, polygenic risk is present before diagnosis, whereas clinical disease emerges later in life. Among children or adolescents, high type 2 diabetes PRS without current diabetes may therefore reflect age-dependent non-expression. In adults, it may reflect early dysglycemia below diagnostic thresholds (e.g., prediabetes), or modifiable risk in which lifestyle and metabolic context influence progression (Guo et al. 2025). For this reason, high-PRS unaffected individuals are best viewed initially as candidates for delayed onset, reduced penetrance, or protective processes rather than as established examples of resilience. Repeated clinical assessment, age-aware analysis, exposure data, and biomarkers can help distinguish persistent discordance from temporary mismatch.

### Conditional and stratified GWAS

PRS can be incorporated into association analysis through regression adjustment, stratified analysis, or interaction modeling. These approaches overlap in practice. Stratification can condition on PRS by estimating the association of interest within PRS-defined groups, so that each estimate is obtained among individuals with similar measured polygenic background. We separate the sections here by implementation: in PRS conditioning, the score is included as a covariate in a regression model; in PRS stratification, the score is used to define analytic subgroups or comparison sets. Stratified analyses may be used to control polygenic background, obtain subgroup-specific estimates, or evaluate heterogeneity across PRS levels. Heterogeneity can then be assessed descriptively through stratum-specific estimates or formally through interaction terms.

#### Conditioning on PRS

Conditioning on PRS treats the score as a background covariate. The aim is to account for additive common-variant burden already represented by prior GWAS, thereby sharpening signals not already tagged by the score (Chatterjee et al. 2016). In practice, this is usually implemented by including PRS as a covariate in regression-based association models, such as logistic regression for binary traits or linear regression for quantitative traits, together with standard adjustment variables including ancestry principal components, sex, age, and study-specific technical covariates. The incremental contribution of PRS can be assessed by comparing otherwise identical models with and without PRS. For example, a trans-ethnic Alzheimer’s disease risk study compared a non-genetic-covariate model with a full genomic informed risk assessment (GIRA) model including PRS to evaluate the added predictive value of PRS relative to other covariates (Sleiman et al. 2023). Resulting associations may therefore be more specific for modifiers, subgroup effects, or mechanisms not well tagged by the score itself.

More flexible implementations are also possible. PRS may be modeled as a continuous covariate, categorized into strata, or entered with nonlinear terms if the relation between polygenic burden and phenotype is not well approximated by linearity on the chosen scale. When PRS is entered as a covariate, it is commonly standardized within the analytic sample or within an ancestry-matched reference set to mean 0 and standard deviation 1. Standardization makes effect estimates easier to compare across studies and scoring methods and reduces scaling problems when interaction or nonlinear terms are included. If PRS is categorized into quantiles or strata, cut points should be defined consistently and, ideally, with reference to the target population or an appropriate external population. Interaction terms between PRS and tested variants can also be examined when the question concerns effect modification by polygenic background, although such models require careful interpretation and substantially greater sample size. Over-adjustment and proximal contamination remain concerns when tested loci, or their linkage disequilibrium proxies, contribute to the score. Leave-one-chromosome-out or leave-local-region-out construction is therefore often needed.

Conditioning is most reliable when PRS is constructed independently of the association test being performed, so that the adjusted model does not import inflation from score derivation. Cross-fitting or sample splitting can reduce this problem when fully external summary statistics are unavailable. Even with proper separation, conditioning removes only the part of background risk measured by PRS.

PRS covariate adjustment has also been examined in psychological causal-association studies, where the score is used to account for measured genetic propensity rather than as the primary predictor (Uddin et al. 2022). PRS adjustment accounts for measured polygenic propensity, but unmeasured genetic confounding can remain. GSens addresses this problem as a sensitivity analysis, combining observed attenuation after PRS adjustment with heritability-based scenarios to estimate the extent to which residual genetic confounding could explain an exposure-outcome association (Pingault et al. 2021). PENGUIN takes a different route by modeling polygenic confounding through variance components, providing a framework for genetic confounding control that does not rely solely on a measured PRS and can be applied with GWAS summary statistics or sample overlap (Zhao et al. 2024). These methods provide ways to evaluate genetic confounding that remains after ordinary PRS covariate adjustment.

#### Stratification by PRS

Stratification partitions the cohort into risk strata and asks whether associations differ across them. Strata are usually defined by PRS quantiles after PRS has been standardized or otherwise calibrated within the relevant analytic population. A locus that is modest or undetectable in pooled analysis may become clearer within a more homogeneous stratum, or within a subgroup where the relevant pathway is more exposed.

The choice between conditioning and stratification depends on the question. If the goal is to test whether a locus is associated with disease after accounting for measured polygenic background, PRS can be included as a covariate in the full-sample model. If the goal is to ask whether a locus is specific to a low-PRS, average-PRS, or high-PRS disease context, stratified analysis is more appropriate. For example, a study searching for rare or noncanonical mechanisms may focus on low-PRS cases, whereas a study testing whether an association is general across the full disease population may prefer conditioning on PRS in the full cohort.

#### Interaction and heterogeneity

Differences across PRS strata should not be interpreted too quickly as biological interaction. Statistical interaction on the modeled scale is not equivalent to biological interaction, because an interaction term may arise from the choice of scale, ascertainment, or subgroup definition rather than from direct mechanistic interplay between genetic effects. A subgroup-specific signal may reflect true dependence on background risk, but it may also reflect threshold effects, altered case composition, phenotype heterogeneity, scale differences, or changes in detectability after stratification (Zhou et al. 2021). Stratified association is therefore evidence of heterogeneity, not automatic proof of genotype-by-PRS interaction.

The practical message is that stratification changes who is being compared with whom. That change can be informative, but it can also alter case composition and the probability of seeing a given signal.

#### Local and global background

A single global PRS is not always the most relevant background. In some settings, pathway-specific, cell-type-specific, age-specific, sex-specific, ancestry-aware, or other context-specific scores may align more closely with the mechanism under study (Jamialahmadi et al. 2024; Schork and Elman 2023; Westerman et al. 2025). The broader literature therefore supports movement from one universal background toward multiple biologically relevant backgrounds. However, these scores should be constructed and interpreted carefully, because increasing biological specificity can also increase multiple testing, reduce portability, and reduce power when strata become small. A separate caution concerns the application of scores developed in well-studied populations to understudied populations, including trans-ethnic PRS applications, where genetic factors (allele frequencies, linkage disequilibrium, imputation quality) and modifying factors (environment and ascertainment) may differ.

### Residual phenotypes

Residual phenotype analysis models a trait on PRS and studies what remains after the PRS-associated component has been removed. This is most straightforward for quantitative traits, where polygenic background can be modeled directly on the observed scale before analyzing the remaining variation. Closely related designs adjust quantitative traits or downstream association models for polygenic background before searching for additional rare-variant, environmental, or molecular signals. For binary outcomes, interpretation is more difficult because the residual depends on the link function, prevalence scale, and ascertainment scheme.

#### Residualization as a scientific operation

Residualization changes the outcome being analyzed. The raw phenotype reflects all contributing sources of variation together. The PRS-adjusted phenotype reflects what remains after the fitted PRS component has been removed. This does not create a non-genetic phenotype. It creates an outcome in which one modeled source of common-variant risk has already been accounted for. This can be useful when the common-variant signal is already reasonably well characterized and the next question concerns what the fitted model does not explain (Zhou et al. 2021). Residualization does not make the biology inherently cleaner. It narrows the question being asked.

#### What residuals can contain

Residuals may contain environmental effects, inherited susceptibility not captured by standard PRS, gene-environment interplay, developmental timing, transient biological states, treatment effects, or measurement error. They may also reflect model misspecification, phenotype misclassification, poor PRS portability, or mismatch between the training GWAS and the target sample (Choi et al. 2020; Pingault et al. 2022). A residual phenotype is therefore useful as a discovery target but is not self-interpreting. Its value comes from redirecting attention to variation not already accounted for by the fitted PRS, not from making that remaining variation automatically causal or mechanistically clean.

#### Over-residualization and biological amputation

Residualization can also remove meaningful biology. If PRS is correlated with the pathway under study, aggressive adjustment may subtract mechanistically relevant signal rather than merely background. This is especially important when downstream phenotypes, molecular profiles, or clinical states partly reflect the same inherited risk summarized by the PRS. Gene-environment correlation, selection, and collider structures further complicate interpretation (Pingault et al. 2022). For binary traits, interpretation is further complicated because the residual depends on modeling choices and case ascertainment.

## Use cases of PRS-informed design

The value of PRS-informed design is especially clear in settings where assays are expensive, outcomes are infrequent, or follow-up is resource-intensive. In these contexts, PRS can be used to define who is selected for sequencing, how omics comparisons are organized, which longitudinal cohorts are enriched, how etiologic heterogeneity is characterized, or which trial subgroups are prioritized (Table 2). This section highlights representative use cases in which PRS functions as a practical design variable rather than as the primary endpoint.

**Table 2:** Matching PRS-informed designs to research applications

Research application	Most useful PRS-informed design	Rationale
Exome or genome sequencing	Low-PRS affected cases; PRS-guided sequencing; two-direction sampling	Enriches for rare-variant yield, uncaptured inherited risk, and informative mismatch
Deep phenotyping	PRS tails; discordance sampling	Sharpens contrast for molecular and clinical profiling
Multi-omics	Residual phenotype analysis; discordance-informed profiling; PRS-stratified molecular analysis	Redirects analysis toward state biology or variation beyond PRS-captured risk
Prevention cohorts	High-PRS tail sampling; high-PRS unaffected follow-up	Increases expected event rate and supports assessment of delayed onset or reduced penetrance
Characterization of etiologic heterogeneity	Stratified GWAS; context-specific PRS; multi-trait or pathway-specific PRS	Reveals subgroup-specific architecture diluted in pooled analyses
Treatment-response studies	PRS-stratified analysis; endpoint-specific PRS; residual phenotype analysis	Tests whether response differs across polygenic background while distinguishing susceptibility from treatment-response endpoints

### PRS-guided exome and genome sequencing

Sequencing is a clear use case for PRS-informed design because rare and common sources of risk are not distributed uniformly across patients. Low-PRS affected individuals are often prioritized because the dominant common-variant model explains less of their disease risk, making them informative for rare pathogenic variants or mechanisms not well captured by that model. In a large population-based sequencing analysis, affected individuals with low PRS were more likely to carry rare pathogenic variants (Lu et al. 2021). This provides a practical cost-efficiency rationale that lower-cost genome-wide genotyping can be used first to identify affected individuals in whom exome or genome sequencing may have higher rare-variant yield.

PRS can also reduce background heterogeneity. Restricting analysis to a narrower PRS range can make individuals more comparable with respect to measured polygenic burden. That does not remove all confounding, but it can reduce dilution from broad susceptibility differences. A low-PRS case series, for example, is not simply a “weaker genetic” sample. It is a sample depleted of one known source of common-variant risk, which may make alternative mechanisms easier to detect.

Recent work in rare neurodevelopmental disorders also shows that common and rare risk can co-occur in structured ways, so PRS-guided sequencing is better viewed as a way to enrich mechanistically informative samples than as a simple common-versus-rare contrast (Huang et al. 2024). Combined use of PRS and family history may enrich inherited susceptibility more effectively than either measure alone because family history may capture rare variants, shared environment, or other familial influences not represented in current PRS. Conversely, individuals with low PRS despite positive family history may be especially informative exceptions, suggesting familial risk that is not well captured by the present common-variant model. For phenotypes in which family history is incompletely reported, including schizophrenia and bipolar disorder, low PRS and negative family history should be interpreted cautiously because stigma or underdiagnosis may obscure familial aggregation despite high heritability (Henderson et al. 2013).

Other sequencing use cases follow the same logic. Sequencing high-PRS cases may help identify combinations of common and rare burden associated with greater penetrance or severity, whereas sequencing high-PRS unaffected individuals may support studies of buffering alleles, delayed penetrance, or protective biology. PRS may also help explain phenotype differences among carriers of the same rare pathogenic variant (Darst et al. 2021). Hybrid models that combine common-variant PRS with rare-variant burden already improve stratification in several settings (Darst et al. 2021; Dornbos et al. 2022). Polygenic score adjustment has been shown to improve power in rare-variant association analyses across quantitative traits (Jurgens et al. 2023).

### Deep phenotyping and multi-omics

Multi-omics provides another practical setting for PRS-informed design. PRS-stratified molecular quantitative trait locus analysis can compare regulatory effects across different risk backgrounds. Large blood eQTL studies show that polygenic scores are associated with broad transcriptional programs, supporting the use of PRS as a biologically meaningful axis for organizing molecular comparisons (Võsa et al. 2021).

Discordance-informed profiling provides a complementary approach. High-PRS unaffected individuals can be selected for studies of delayed expression, compensation, or protection, whereas low-PRS affected individuals can be selected to enrich mechanisms outside the dominant common-variant pathway. At single-cell resolution, projected polygenic burden may also help dissect disease heterogeneity and support profiling strategies beyond standard case-control comparisons (Zhang et al. 2025). In pediatric asthma, for example, we used a PM2.5 sensitivity PRS (sPRS) to compare matched high- and low-sPRS children and identified TGF-β1-SMAD/MAPK-related inflammatory and stress-response programs across immune cell populations (Kelchtermans et al. 2026).

PRS-adjusted molecular analysis is useful when investigators want to study disease expression, treatment-related biology, or exposure-related states after accounting for PRS-captured risk. Residual phenotypes can anchor downstream analysis by directing attention to variation not already captured by known common-variant burden, including rare genetic effects, exposures, molecular states, compensatory processes, and model-related variation.

In environmental and exposome studies, this approach may help separate associations with the overall phenotype from associations with the non-PRS component of that phenotype. Argentieri et al. (2025) showed that environmental exposures contribute to disease and mortality patterns beyond measured polygenic disease risk (Argentieri et al. 2025). Pingault et al. (2022) specifically emphasize that associations among polygenic scores, environmental exposures, and phenotypes can arise through multiple pathways, including gene-environment correlation and structural sources of bias, which is directly relevant to interpretation of residual signals (Pingault et al. 2022).

This caution is especially important in omics analyses, where common-variant risk may already be reflected in transcriptomic, proteomic, or imaging profiles. In the obesity literature, molecular correlates of BMI trajectories reflect both shared etiology and state-dependent biology, with overlapping but non-identical genetic, environmental, and metabolic influences (Drouard et al. 2023). Aggressive residualization may therefore reduce confounding by inherited background, but it can also remove biologically informative signal when that background contributes to the molecular state under study.

Residual phenotypes are most informative when the remaining variation shows reproducible structure rather than random noise. After accounting for common-variant burden, some individuals may cluster more strongly by inflammatory or metabolic features, whereas others may be distinguished by developmental, behavioral, or treatment-related profiles. In disorders influenced by both common and rare variation, the residual component may also help separate individuals whose presentation is more consistent with rare-variant burden or atypical disease pathways (Niemi et al. 2018; Urpa et al. 2024). When such patterns recur across cohorts, assays, or clinical subgroups, they support biologically meaningful heterogeneity and can help identify mechanisms obscured in the unadjusted phenotype.

### Etiologic heterogeneity and stratified association

PRS-stratified analysis is useful for characterizing etiologic heterogeneity because it asks whether disease architecture differs across modeled common-variant backgrounds. PRS-stratified GWAS can help separate biologically distinct components of a clinically defined trait. If loci emerge only among low-PRS cases, they may mark disease forms less dependent on the dominant common-variant pathway. If loci emerge only among high-PRS cases, they may reflect risk-dependent penetrance or downstream amplifiers that matter once background burden is already high. Stratification alone does not prove definitive subtypes, but repeated subgroup-specific signals can point toward mechanistic partitions that pooled analysis obscures.

Type 1 diabetes is a disease-specific example of PRS-stratified analysis. It is often early-onset and has a strong HLA contribution, so PRS-informed stratification may be more informative in this setting than in complex diseases with later onset and weaker common-variant architecture. In this context, low-PRS cases are informative because their disease is less well explained by current PRS models and the canonical autoimmune model. Subgroup-restricted analysis of low-PRS type 1 diabetes cases identified loci not prominent in pooled analyses, including 13 novel loci in one study and association with *DLL1* in another (Qu et al. 2021a, b, c). The *DLL1* association provides an example of decile-based support for a subgroup-specific signal, with the association strongest among individuals with lower PRS rather than uniformly distributed across the full PRS range (Qu et al. 2021c).

Beyond locus discovery, PRS-stratified analysis may also help clarify heterogeneity within broad clinical labels. In clinically broad pediatric diabetes cohorts, it may help separate atypical autoimmune diabetes, monogenic diabetes, or youth-onset type 2 diabetes that share an initial clinical label but differ biologically. This is especially useful in disorders with broad clinical definitions or mixed pathophysiology, such as type 1 diabetes and metabolic dysfunction-associated steatotic liver disease (MASLD) (Jamialahmadi et al. 2024).

The relevant polygenic background is not always best represented by one global score. In some settings, a context-specific PRS defined by sex, age, ancestry, pathway, or another biologically meaningful axis may be more informative than a pooled score. For example, sex-stratified analyses in type 1 diabetes identified male-specific and female-specific risk loci, showed that sex-specific PRS may outperform pooled PRS, and supported sex-specific architecture at the level of cell-type-specific gene expression (Qu and Hakonarson 2025; Qu et al. 2026).

### Prevention cohorts and longitudinal follow-up

Prevention cohorts and longitudinal follow-up studies provide another natural use case for PRS-informed design. This is especially useful before disease onset. For late-onset disorders, phenotype-based ascertainment is delayed and vulnerable to censoring. PRS is fixed by genotype and can be assessed before disease onset, allowing early enrichment for follow-up, biomarker discovery, or prevention studies (Khera et al. 2018).

High-PRS unaffected individuals are informative in this setting. They are relevant for longitudinal studies of delayed onset, reduced penetrance, resilience-related biology, or early biomarkers. However, high-PRS unaffected status should be interpreted cautiously, especially in age-dependent or slowly progressive disorders, because some individuals classified as unaffected will later become cases. Studies in schizophrenia and late-onset Alzheimer’s disease show how high-PRS unaffected individuals can be used to study resilience-related biology, while also illustrating the need for careful interpretation (Hess et al. 2021; Hou et al. 2022). Repeated clinical assessment, age-aware analysis, exposure data, and biomarkers can help distinguish persistent discordance from temporary mismatch.

### Trial design and treatment-response studies

Trials provide a further use case for PRS-informed design. In prevention or event-driven studies, PRS-based enrichment may help identify individuals more likely to experience the outcome of interest. This enrichment logic is consistent with the broader use of PRS in risk prediction, screening, and precision prevention (Wang et al. 2021). In treatment-response studies, PRS-stratified analysis can test whether response differs across polygenic backgrounds (Pardiñas et al. 2022). In mechanism-oriented trial design, PRS may help distinguish biology already captured by common-variant risk from biology that remains potentially modifiable.

This distinction is particularly relevant when an intervention acts downstream of inherited susceptibility. Low-PRS affected individuals may help identify disease processes operating outside the dominant common-variant pathway, whereas high-PRS unaffected individuals may be useful for studying buffering or compensatory processes. Residual phenotypes may also serve as outcomes when the goal is to evaluate treatment effects on biology that remains after PRS-captured risk has been accounted for.

A key limitation is that PRS usually indexes disease susceptibility, whereas many trial endpoints concern disease course after onset. A susceptibility PRS may enrich a trial for individuals likely to develop disease, but it should not be assumed to index progression, complications, survival, or other post-onset outcomes. Recent evidence shows limited overlap between genetic effects on disease susceptibility and disease-specific mortality, with susceptibility PGS showing weak prediction of disease-specific mortality (Yang et al. 2025). Progression-oriented trials may require genetic instruments or scores derived from progression-relevant endpoints rather than disease susceptibility.

Important limitations also apply to treatment-response applications. A PRS for disease susceptibility should not be assumed to predict treatment response. This distinction is important in major depressive disorder, where placebo response is often substantial and can reduce separation between active treatment and control arms (Jones et al. 2021). Pharmacogenomic predictors such as CYP2D6, CYP2C19, and CYP2B6 metabolizer status may be better suited than PRS for addressing drug metabolism and dosing of antidepressants (Bousman et al. 2023).

## Biases, failure modes, and ethical risks

PRS-based design turns polygenic background into a measurable study variable, which may create new failure modes. These risks are structural rather than peripheral. Sampling, conditioning, and residualization can convert PRS imperfection into analytic distortion when the score is unstable, non-portable, or entangled with selection.

### Data leakage and overfitting

If the same data contribute to PRS derivation and downstream analysis, this creates data leakage. The score may carry overfitted signal into the follow-up study. However, this is an analytic-design flaw rather than a limitation of PRS itself. Sample overlap inflates apparent informativeness, and winner’s curse can exaggerate the importance of variants used to define the background itself (Ellis et al. 2024; Shi et al. 2016). This is not only a problem for prediction accuracy. It affects any design that samples on PRS, conditions on PRS, or defines residual phenotypes using an in-sample score.

Residualization is especially vulnerable. Regressing a phenotype on an in-sample PRS and then treating the residual as a clean discovery target may distort downstream associations, because part of the apparent remainder may reflect overfit subtraction rather than genuine biology. External PRS, cross-validation, cross-fitting, and clear separation between score construction and downstream analysis should therefore be treated as design requirements.

### Collider structures

Collider bias is a distortion created when a study selects patients on a characteristic influenced by more than one upstream factor. Once that selection occurs, variables that were independent in the source population can appear associated. Conditioning on disease status, sample selection, or discordance can therefore induce spurious associations among genetic, environmental, and phenotypic variables that were not associated in the source population (Akimova et al. 2021; Munafò et al. 2018). This is especially relevant when PRS is used to define atypical cohorts such as low-PRS cases or high-PRS unaffected controls, and in disease-progression settings where selection depends on having remained free of an outcome despite elevated risk. Such designs can be valuable for discovery, but effect estimation within the selected sample requires explicit attention to selection structure (Mitchell et al. 2023). PRS-informed enrichment may improve discovery efficiency, but associations observed within the selected sample should not be interpreted automatically as unbiased causal effects, because selection itself can distort relationships among variables.

### Transportability

Current PRS are usually more accurate in populations genetically similar to the GWAS from which they were trained, and performance can also vary by age, ascertainment, phenotype definition, and technical platform (Duncan et al. 2019; Martin et al. 2019). A score that seems acceptable for risk ranking may still perform poorly when used to assign tails, define discordance, or residualize phenotypes.

This matters especially for design-based use because misclassification changes who gets sequenced, deeply phenotyped, or recruited for follow-up. Design-based use of PRS may therefore amplify health disparities even more sharply than clinical interpretation alone if the score captures less of true background risk in some groups than in others (Guo et al. 2025; Lennon et al. 2024). Portability should be evaluated with the intended design use in mind, not only with standard predictive metrics.

### Temporal instability

Discordance is often time-dependent. For age-dependent disorders, high-PRS unaffected status is not equivalent to resilience, because some individuals will simply have later onset or slower progression rather than true protection. This is especially important in late-onset neurodegenerative disease, where latent pathology may be present long before diagnosis and progression rates can vary substantially across individuals (Eissman et al. 2023; Hou et al. 2022).

The implication is straightforward. Claims about resilience, protection, or delayed penetrance require age-aware interpretation and, when possible, longitudinal reassessment of both onset and progression. Without follow-up, high-PRS unaffected status is useful as an enrichment criterion but weak as a biological label.

### Ethical vocabulary

Terms such as high risk, resilient, protected, and noncanonical can turn probabilistic differences into personal labels. In recruitment and consent, this may mislead participants about inevitability, exceptionality, or biological certainty. The same warning that applies to clinical use applies to design-based use as well. This is especially important for stigmatized conditions, including psychiatric disorders, where risk labels may amplify social stigma, discourage disclosure, or be misread as deterministic statements.

High-PRS unaffected individuals should not be described as genetically resilient without longitudinal evidence. Low-PRS cases should not be framed as biologically exceptional in ways that imply diagnostic certainty or etiologic purity. Deterministic language is scientifically inaccurate and ethically unnecessary. PRS estimates one modeled component of risk; it does not define destiny.

## Future directions

PRS have long been used in contexts beyond individual risk prediction, including genetic epidemiology and discovery-oriented analyses. Once interpreted as an estimate of known common-variant risk, PRS can be used not only as a predictive output, but also as a design variable for sampling, subgroup definition, and analytic comparison. Its value in this setting lies in making one measured component of genetic background explicit, while recognizing that PRS does not replace family history or established genetic-epidemiologic designs.

### Iterative discovery loops

The next phase of PRS-based design is likely to be iterative rather than static. Large cohorts generate GWAS summary statistics. Those statistics produce PRS. PRS then guides sampling for sequencing, deep phenotyping, and molecular profiling. The resulting discoveries refine risk models and improve the next round of ascertainment. Prediction and discovery are not always separate stages. They may form a loop when score construction and downstream analysis are kept analytically independent. Such designs require careful separation between PRS derivation and follow-up analysis to avoid data leakage and overfitting.

### Beyond scalar PRS

A single scalar score is often too crude for the biology under study. Future designs will likely rely more on contextual or modular PRS reflecting pathway, cell type, ancestry, age, sex, or exposure-specific risk. The point is not only better prediction, but better alignment between the score and the mechanism under study (Guo et al. 2025; Jamialahmadi et al. 2024; Westerman et al. 2025). These more specific scores can also reduce sample size, increase multiple-testing burden, and reduce portability across cohorts.

A related next step is the use of multi-trait and pathway-specific PRS to model risk more realistically. Many diseases arise within networks of genetically correlated traits rather than in isolation. A single disease-specific PRS may therefore represent only part of the relevant background. Multi-trait PRS captures shared risk across related phenotypes, whereas pathway-specific PRS localizes burden to particular biological systems. Multi-trait PRS may be particularly useful when related phenotypes share substantial genetic risk, as in schizophrenia and bipolar disorder. Richards et al. used genomic structural equation modeling of schizophrenia, bipolar disorder, and major depressive disorder GWAS to derive PRS components representing common and disorder-differentiating liabilities, supporting potential use for patient stratification and precision therapeutics development (Richards et al. 2022). In study design, these approaches may refine discordance, improve subgroup definition, and identify loci, biomarkers, or treatment effects that emerge only within particular trait networks or pathways. This may be especially valuable in therapeutic and trial settings, where pathway-specific PRS centered on the drug target or perturbed biology could improve patient enrichment and clarify heterogeneity in treatment response. Clinical use of such scores requires endpoint-specific validation and should not be inferred from discovery performance alone.

### PRS-guided adaptive cohorts

Cohort design may also become adaptive. Instead of fixing recruitment thresholds once, studies may update tail definitions, oversample underrepresented discordance classes, and revise enrollment rules as models improve. A cohort need not be a passive repository. It can be designed to learn from its own yield.

This may be especially useful for resource-intensive assays and long-term follow-up studies. If high-PRS unaffected individuals become scarce in an aging cohort, recruitment may shift toward that class. If low-PRS affected cases prove especially informative for sequencing, thresholds may be updated to deepen that subgroup. Such adaptive approaches require attention to threshold instability, changing recruitment probabilities, and context-dependent PRS performance across technical settings and populations (Guo et al. 2025; Ho et al. 2025).

### PRS-informed epidemiologic studies

Traditional epidemiologic contrasts compare cases and controls, exposed and unexposed groups, or responders and non-responders, often with incomplete control of inherited background. PRS provides a partial but measurable estimate of common-variant risk. It does not solve confounding on its own, and it does not replace family history, which captures shared genetic, environmental, and social factors not represented in PRS. Rather, PRS can make one component of inherited background more visible and allow some comparisons to be organized more explicitly around measured common-variant risk.

The practical meaning is straightforward. A high-PRS unaffected individual and an average-PRS unaffected individual are not interchangeable controls with respect to modeled common-variant risk. A low-PRS case and a typical case are not interchangeable cases with respect to the extent to which disease is explained by the fitted common-variant model. Once inherited background is measured, investigators can compare individuals with more explicit control of common-variant risk and ask whether observed differences are consistent with that background or instead point to other contributors, such as rare variants, exposures, or disease subtypes. These comparisons remain descriptive unless supported by an appropriate causal design.

**Table 3:** Operational recommendations for PRS-informed study design

*Use external, cross-validated, or cross-fitted PRS*.
Avoid data leakage, overfitting, winner’s curse, and sample-overlap bias.
*Avoid proximal contamination.*
In PRS-conditioned GWAS, exclude the tested region from the conditioning score.
*Validate PRS in the target population*.
Tail assignment and discordance depend on ancestry, ascertainment, allele harmonization, technical platform, and score portability.
*Pre-specify distribution-defined cutoffs*.
Use external or target-population reference distributions when defining low-, intermediate-, or high-PRS groups. Internally defined quantiles should be interpreted as sample-relative strata.
*Treat discordance as time-dependent*.
High-PRS unaffected status may reflect delayed onset, early subclinical disease, slower progression, competing risks, or misclassification.
*Separate enrichment from causal inference*.
Informative sampling does not by itself support unbiased causal inference or establish mechanism.
*Match design to question*.
Tail enrichment for ascertainment; discordance for informative phenotype-PRS deviation; stratification for heterogeneity; conditioning for measured polygenic background; residuals for uncaptured variation.
*Account for power in stratified designs*.
Small cohorts, clinical trials, or two-dimensional sampling may require broad strata, pre-specified contrasts, or continuous modeling rather than full stratification.
*Interpret residuals cautiously*.
Residual phenotypes are model-dependent, especially for binary traits.
*Replicate subgroup findings externally*.
Confirm PRS-defined signals in independent cohorts.

Table 3 summarizes practical recommendations for implementation. Used wisely, PRS can improve sampling, sharpen subgroup definition, support rare variant discovery, and help map heterogeneity within defined risk backgrounds. PRS should be interpreted as one measured component of inherited risk, not as a replacement for family history or a self-sufficient explanation of disease biology.

## Data Availability

No datasets were generated or analysed during the current study.
